# Pot experimental trial for assessing the role of different composts on decontamination and reclamation of a polluted soil from an illegal dump site in Southern Italy using *Brassica juncea* and *Sorghum bicolor*

**DOI:** 10.1007/s11356-023-31256-3

**Published:** 2023-12-08

**Authors:** Martina Mazzon, Nicole Bozzi Cionci, Enrico Buscaroli, Daniele Alberoni, Loredana Baffoni, Diana Di Gioia, Claudio Marzadori, Lorenzo Barbanti, Attilio Toscano, Ilaria Braschi

**Affiliations:** Department of Agricultural and Food Sciences - Alma Mater Studiorum University of Bologna, (BO), Bologna, Italy

**Keywords:** Potentially toxic elements (PTE), Organic contaminants, Persistent organic pollutants (POPs), Soil microbiota, Soil biochemistry

## Abstract

A pot experiment was carried out to evaluate the remediation potential of *Brassica juncea* and *Sorghum bicolor* in the decontamination of soil polluted with heavy metals such as copper, lead, tin, and zinc along with polychlorinated biphenyls, polycyclic aromatic hydrocarbons, and heavy hydrocarbons. Two composts obtained from different composting processes were tested as biostimulating agents. At the end of the trial, the effect of plant/compost combinations on soil microbial composition, contaminant removal, biochemical indicators, and plant biomass production was determined. The results highlighted that compost addition improved plant biomass despite slowing down plants’ removal of organic and inorganic contaminants. In addition, compost partially enhanced the soil biochemical indicators and modified the relative abundance of the rhizosphere microorganisms. *Sorghum* showed better mitigation performance than *Brassica* due to its higher growth. The soil fertility level, the choice of plant species, and microbial richness were found fundamental to perform soil remediation. In contrast, compost was relevant for a higher crop biomass yield.

**Graphical abstract:**

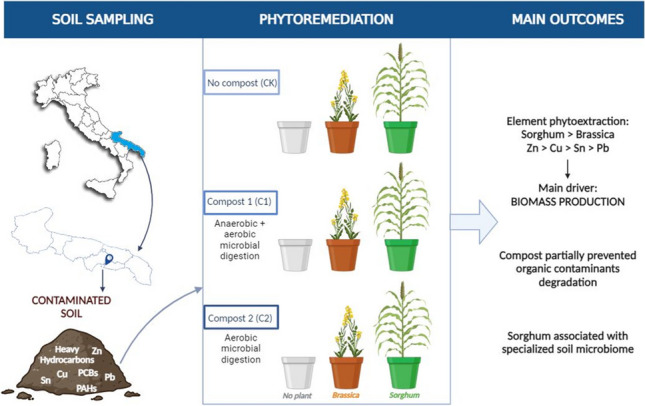

**Supplementary Information:**

The online version contains supplementary material available at 10.1007/s11356-023-31256-3.

## Introduction

Steel plants are recognized worldwide as a source of heavy environmental pollution (Zeng et al. 2009) and of contamination of the surroundings. As multiple studies have reported (Emili et al. 2016; Mali et al. 2017), air, water, and soil were found to be contaminated with hazardous pollutants (Wang et al. 2016; Sun et al. 2019; Odabasi et al. 2010), such as heavy hydrocarbons, potentially toxic elements (PTE), polychlorinated biphenyls (PCB), dioxin-like compounds, and polycyclic aromatic hydrocarbons (PAH). Environmental remediation of contaminated lands around the plants involves intensive or extensive remediation techniques. While intensive treatments are undoubtedly effective in rapid contaminant destruction or immobilization, they are deemed inadequate for disadvantaged areas suffering from mild contamination, as their costs outweigh marginal benefits. Phytoremediation technologies may efficiently provide multiple beneficial outputs (e.g., energy crop production, urban renewal, and creation of ecological buffer areas), besides the primary function of mitigating soil contamination (Chandra et al. 2017; Guidi Nissim and Labrecque 2021) and improving landscape quality.

Phytoremediation shows great effects in soil PTE decontamination (Ali et al. 2013) and in the degradation of organic pollutants. More specifically, plant growth can induce biodegradation at the rhizosphere level, through the release of secondary metabolites and root exudates which play a key role as microbial biostimulating agents (Newman and Reynolds 2004; Dudášová et al. 2012; Toussaint et al. 2012). Indeed, plants establish mutual beneficial interactions with specific bacterial populations that promote nutrient uptake and enhance stress tolerance against pollutants, decreasing their phytotoxicity (Abhilash et al. 2016; Ma et al. 2011). For example, multiple microbial groups are capable of metabolizing PCB using biphenyl as a primary substrate (Kurzawova et al. 2012; Leigh et al. 2007).

Soil microbial degradation of persistent organic pollutants (POPs), and more generally organics, can be facilitated through external inputs. Supplying contaminated soils with compost, manure, or other organic amendments could be of pivotal value to soil remediation (Ventorino et al. 2019), because it improves soil physical properties (Lwin et al. 2018; Masciandaro et al. 2013; Sciubba et al. 2015) and provides cheaper nutrients to plants and microorganisms if compared to mineral fertilizers (Matsumura et al. 2015). Additionally, organic soil amendments may be locally produced from renewable sources, thus reducing pollution while enhancing the local circular economy. Economic sustainability plays a key role in determining the outcomes of a soil remediation project. To this extent, growing crops finalized to energy production (e.g., biomass, biodiesel) in phytoremediation plots may represent a valid opportunity to measure phytoremediation efficiency and the costs-benefits evaluation.

Among specialized crops, the Brassicaceae family includes one species (i.e., *Brassica juncea*) that is considered effective in PCB degradation (Pino et al. 2019; Terzaghi et al. 2019) and in PTE extraction from contaminated soils (Awad et al. 2020; Jeyasundar et al. 2021). Another specialized crop is *Sorghum bicolor* belonging to the Poaceae family (Lima et al. 2019; Napoli et al. 2019), characterized by the excellent ability of its root system to explore the substrate, and the very high biomass yield.

The present work investigates the short-term phytoremediation efficacy of *Brassica juncea* and *Sorghum bicolor* in the context of a marginal area contaminated with both organic (PAH, PCB, and heavy hydrocarbons) and inorganic (Cu, Pb, Sn, and Zn) pollutants, in the presence of two composts obtained from different composting processes. Pot trial was established with contaminated soil collected from a former illegal dump site in the Mar Piccolo area (Taranto, Italy) which was placed under government seizure due to its high contamination level. This study aims at highlighting the fundamental aspects and/or issues that need to be considered in the on-field phytoremediation design.

## Materials and methods

The spot chosen for soil sampling (40°28′4.45″N, 17°18′13.51″E) is located in the “Ex-campo Cimino-Manganecchia” area, near the Mar Piccolo Sea lagoon, northeast of the city center of Taranto, used for decades as illegal dump site and in the vicinity of the large ironworks in operation since the 1960s. The soil was a Cambisol (WRB-IUSS 2015) with a pH of 8.74, an electrical conductivity of 363 μS cm^−1^, and a total nitrogen and organic carbon respective content of 0.24% and 1.98%, as already described by Ancona et al. (2017). In Table 1 are reported the trace element and the organic contaminant content present in the soil at the time of sampling. Approximately 150 kg of bulk soil was collected and transported to the laboratory for preparation and chemical characterization. The whole amount was sieved at 2 mm, homogenized, and stored at 4 °C.

**Table 1:** Trace element and organic contaminants content in the soil at time of sampling. Results are reported in mg over kg of dry soil (ds) and compared with the limits of the Italian law for the green areas (D.Lgs. 152/06)

	Soil at sampling	*LOQ	D.Lgs. 152/06 Contamination tresholds
Trace element (mg kg_ds_^−1^)			
As	3.26	0.0068	20
Sb	1.17	0.02	10
Be	0.316	0.001	2
Cd	0.690	0.001	2
Co	nd	nd	20
Cr (total)	23.5	0.002	150
Hg	<LOQ	0.002	1
Ni	17.0	0.003	120
Pb	160	0.006	100
Cu	105	0.001	120
Tl	<LOQ	0.004	1
Se	<LOQ	0.007	3
Sn	18.7	0.01	1
V	17.0	0.003	90
Zn	326	0.0007	150
Organic contaminants			
PAH (mg kg_ds_^−1^)	1.80	0.01	10
Heavy hydrocarbons (mg kg_ds_^−1^)	105	5	50
Sum of dioxins and furans (ng I-TEQ** kg_ds_^−1^)	5.90	0.1	10
PCB (mg kg_ds_^−1^)	0.165	0.006	0.06

*LOQ = limit of quantification

**I-TEQ = International standard Toxic Equivalent of dioxins and furans

### Phytoremediation trial

**Fig. 1:**
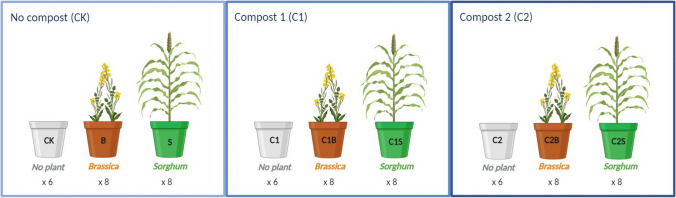
Schematic representation of the nine experimental treatments focused in this work. [CK] no compost; [C1] compost 1; [C2] compost 2; Brassica [B]; Sorghum [S]

The experimentation layout combined two factors: “plants” and “composts” for a total of 66 experimental units (Fig. 1). The nine treatments resulted from the combination of the three compost options (CK, C1, and C2) with the three plant options (none, Brassica, and Sorghum); eight replicates were established in the presence of plants and six for the treatments without plant.

The two organic materials originated from different composting processes were selected. Compost 1 (C1) was obtained through a combined anaerobic and aerobic microbial digestion of vegetable residues. Compost 2 (C2) was obtained through a single aerobic microbial digestion cycle of vegetable residues. A detailed physico-chemical characterization is shown in Table S1, as a supporting information. Compost C1 and C2 had a pH of 6.86 and 6.44, an organic carbon (C) content of 33 and 36%, and a total nitrogen (N) content of 2.31 and 1.89% on dry mass, respectively. The different C to N ratio and germination index were fundamental in compost selection as potential indicators of their different tendency to be degraded in the soil. Compost C1 or C2 was added to soil pots at a concentration of 1.2% (dry mass ratio). The soil used was the contaminated soil sampled from the “Ex-campo Cimino-Manganecchia” area and previously described. Control pots were also prepared without the addition of organic material (CK). In all cases, the soil mass was 1.5 kg per pot. A preliminary sampling of the three substrate combinations (CK — soil from Taranto, C1 — soil with 1.2% Compost 1, and C2 — soil with 1.2% Compost 2) was carried out at time zero (T0).

Commercial seeds of *Brassica juncea* (L.) Czern. (B) (Red Mustard cultivar supplied by Suba Seeds Co., Longiano, FC, Italy) and *Sorghum bicolor* (L.) Moench. (S) (Bulldozer hybrid supplied by KWS, Monselice, PD, Italy) were planted in small cells filled with commercial peat for gardening, and subsequently wet. After 2 days (d) of incubation, the seeds germinated; 5 d after emergence (April 23, 2020), seedlings were transplanted into experimental pots. Pots were put in a growth chamber at 20 °C for 7 d and watered with approximately 250 mL of water on the first day.

On May 8, 2020, the pots were relocated outdoors in pseudo-field conditions, in the Agricultural Plant Garden at the Department of Agricultural and Food Sciences (University of Bologna). The pots were isolated from the underlying soil by a polyvinyl chloride layer and protected from rodents by an electro-welded mesh. Water was added at need, especially during July and August. At the end of the trial (August 26, 2020), each experimental pot was dismantled and the soil, root system, and aerial parts were separated. After thorough rinsing, root and shoot samples were dried at 60 °C for 72 hours (h) and milled. Homogenized soil samples were partly stored at 4 °C, partly dried at 105 °C for 24 h before milling, and partly stored at −80 °C until microbial analysis. Samples obtained at the end of the trial were labelled as T1.

### Trace element analysis

Trace element content in soil (before compost addition — see Table 1 — or after experimentation) and vegetable samples was determined by wet acid digestion. Adaptations of International Organization for Standardization (ISO) methods 12914:2012, 22036:2008 were used for soil and vegetal samples. Briefly, 250 mg of soil samples were digested adding 6 mL of fuming hydrochloric acid (37% for trace analysis, Honeywell, Fluka) and 2 mL of nitric acid (65% HNO_3_ for trace analysis, Honeywell, Fluka) in polytetrafluoroethylene pressure-resistant vessels. Milled vegetable samples (250 mg) were digested using 6 mL of nitric acid and 2 mL of hydrogen peroxide (for electronic use, Honeywell, Fluka). Microwave-assisted digestion occurred for 10 min, and the total cycle duration was 60 min. Suspensions were filtered through Whatman no. 42 filter paper and analyzed by a Spectro Arcos ICP-OES (Ametek, Germany). As an analytical quality control, the determinations were done in duplicate. Moreover, the analysis of a blank sample and a reference material were performed in every analytical batch.

### Organic contaminants analysis

Organic contaminants analyses were performed by CSA S.p.A. group (Rimini, Italy) external laboratory, ISO 11725 certified. Soil samples were extracted by ultrasonic-assisted solvent extraction (US EPA 3550C 2007). PAH determination on soil extracts was performed by gas chromatography coupled with a mass spectrometry detector (US EPA 820E 2018). Non-halogenated heavy hydrocarbon content (> 12 carbon atoms) determination was performed on extracts by gas chromatography coupled with a flame ionization detector (EPA 8015C 2007). Lastly, PCB content of soil extracts was quantified by gas chromatography coupled with electron capture detector.

### Soil chemical and biochemical indicators

Soil pH was measured in ultrapure water (ISO 10390:2005). Soil total organic carbon (SOC) and total nitrogen (TN) were analyzed by a Flash 2000 elemental analyzer (Thermo Fisher Scientific). Soil microbial biomass carbon (MBC) and nitrogen (MBN) were determined using the chloroform-fumigation extraction method (Vance et al. 1987), on potassium sulfate extracts by a TOC - TN Hypertoc Shimadzu elemental analyzer (Shimadzu Corp., Kyoto, Japan). MBC was calculated as the difference between organic C in the fumigated and that in the unfumigated soil extracts. The organic C content of the unfumigated extracts was used as an estimation of soil extractable carbon (DOC, dissolved organic carbon); similarly, MBN and soil extractable nitrogen (TDN, total dissolved N) were calculated (Vance et al. 1987).

The potential β-glucosidase activity (β-glu) was determined following the procedure of Eivazi and Tabatabai (1988). Briefly, 1 g of soil was incubated with *p*-nitrophenyl-β-glucoside (pNG) at 37 °C for 1 h. The *p*-nitrophenol (pN) released was measured at *λ* 400 nm, and the activity was expressed as μg_pN_ g_ds_^−1^ h^−1^. The potential activity of dehydrogenase (Dehy), an intracellular enzyme, was measured according to Von Mersi and Schinner (1991). Released iodo-nitrotetrazolium formazan (INTF) was measured at *λ* 464 nm, and the activity was expressed as μg_INTF_ g_ds_^−1^ h^−1^. The specific soil enzymatic activities (Gil-Sotres et al. 2005; Kandeler and Eder 1993; Trasar-Cepeda et al. 2008) were calculated by dividing the enzymatic activity values by the MBC.

Two biochemical indexes were determined: the microbial quotient (qmic) and the metabolic index (MI). qmic was obtained dividing MBC by the SOC content (expressed as mg_MBC_ g_SOC_^−1^), while the MI was obtained dividing the Dehy by the DOC (expressed as mg_INTF_ mg_DOC_^−1^ h^−1^).

### Microbial analyses of soil pots

Total genomic DNA was extracted from soil samples collected at T0 and T1 by a PowerLyzer PowerSoil Kit (Qiagen, West Sussex, UK) according to the manufacturer’s instruction. The purity of extracted DNA was evaluated measuring the ratio of absorbance at 260 and 280 nm (Infinite®200 PRO NanoQuant, Mannedorf, Switzerland), and the DNA concentration was estimated at 260 nm with a Qubit® 3.0 Fluorometer (Invitrogen, Life Technologies, Carlsbad, CA, USA). Absolute quantification of total bacteria and fungi was done using quantitative PCR (qPCR) in a 10-μL PCR amplification mixture containing 5 μL of Fast SYBR® Green Master Mix (Applied Biosystems, Foster city, CA, USA), optimized concentrations of primers (Table S2, as a supporting material), molecular grade H_2_O, and 2 μL DNA at a concentration of 5 ng/μL.

DNA samples were subjected to Illumina sequencing. The V3–V4 region of 16S rRNA gene was amplified with universal primers suggested by Takahashi et al. (2014) (Table S2) and sequenced. The assays were performed with a 40-μL PCR amplification mixture containing 20 μL of HiFi HotStart ReadyMix (KAPA Biosystems, Woburn, MA, USA), optimized concentration of primers (0.2 μM), and molecular grade H_2_O and 3 μL DNA (5 ng/μL). PCR amplification was performed as follows: activation at 95 °C for 3 min followed by 25 cycles at 95 °C for 30 s, 55 °C for 30 s, and 72 °C for 30 s, followed by a final elongation step at 72 °C for 5 min. PCR products were cleaned using a AMPure beads XP purification system (Beckman Coulter, UK) following Illumina 16S Ribosomal RNA Gene Amplicon instructions. Illumina sequencing adapters and dual-index barcodes were added to amplicons using a Nextera XT index kit (Illumina, SanDiego, CA, USA). The second PCR amplification was performed as follows: 95 °C for 3 min followed by eight cycles of 95 °C for 30 s, 55 °C for 30 s and 72 °C for 30 s, and a final elongation at 72 °C for 5 min. A further cleaning using AMPure beads XP purification system (Beckman Coulter, UK) was performed. Amplicons were quantified using a Qubit® 2.0 Fluorometer (Invitrogen, Life Technologies, Carlsbad, CA, USA) and pooled in an equimolar mode following library preparation and sequenced on the MiSeq platform. The sequencing process was outsourced at Macrogen Inc. (Next Generation Sequencing Division, Seul, Republic of Korea), using a 2 × 300 pair-end protocol.

The resulting 300 bp paired-end reads were assembled using FLASH (Magoč and Salzberg 2011). Further sequence read processing was performed using QIIME ver. 1.9.1 (Caporaso et al. 2010b) and ChimeraSlayer (Haas et al. 2011), including quality filtering based on a quality score > 25 and removal of mismatched barcodes and sequences below length thresholds. Denoising, chimera detection, and clustering into operational taxonomic units (OTUs) (97% identity) were performed using USEARCH version 7 (Haas et al. 2011). OTU sequences were aligned using PyNAST (Edgar 2010), and taxonomy assignment was determined using the SILVA SSU Ref database release 111 (Caporaso et al. 2010a). Biodiversity index analysis was performed using QIIME tools, in particular the script “core_diversity_analysis.py”; the phylogenetic classification of OTUs was carried out with the script “make_phylogeny.py” (fasttree). α-Diversity was evaluated considering Chao1, Observed_OTU, and PD_whole_tree metrics. β–Diversity was evaluated using the “weighted_unifrac” method (Quast et al. 2012).

### Statistical analysis

Data related to plant and soil were checked for normality (Shapiro-Wilk test) and homogeneity of variance (Levene test) and then analyzed with a two-way ANOVA (with plant and compost as factors), followed by Tukey’s HSD test at *p*-value < 0.05 to assess the significance of differences among treatments. The normality and homogeneity of variance of microbial datasets were also checked; statistical significance was evaluated with “glm” (Generalized Linear Model) with the pairwise comparison using “lsmeans” function and with the least significant difference test (LSD test, *p*-value < 0.05) with Bonferroni correction. Pearson’s correlation coefficients (*r*) were also calculated and plotted by using the “ggcorrplot” package. All the statistical analysis of the data was performed using the R environment version 4.2.2 (R core team 2023).

## Results

### Plant biomass

*Brassica* plants produced an average of 4.4 ± 0.2 g and 1.4 ± 0.2 g of dry biomass (db), respectively, for shoots and roots across the three experimental conditions [B], [C1B], and [C2B], with no significant differences among compost treatments. On contrary, *Sorghum* was more affected by the soil conditions, and the shoots produced 15.3 ± 1.4 g [S], 18.2 ± 1.5 in [C1S], and 20.7 ± 0.8 g in [C2S], although no significant differences were detected. Interestingly, the average root biomass followed an opposite trend with respect to shoots with 16.8 ± 3.7 g in [S], 11.3 ± 2.2 g in [C1S], and 9.7 ± 1.2 g in [C2S].

### Trace elements and organic contaminants in soil

**Fig. 2:**
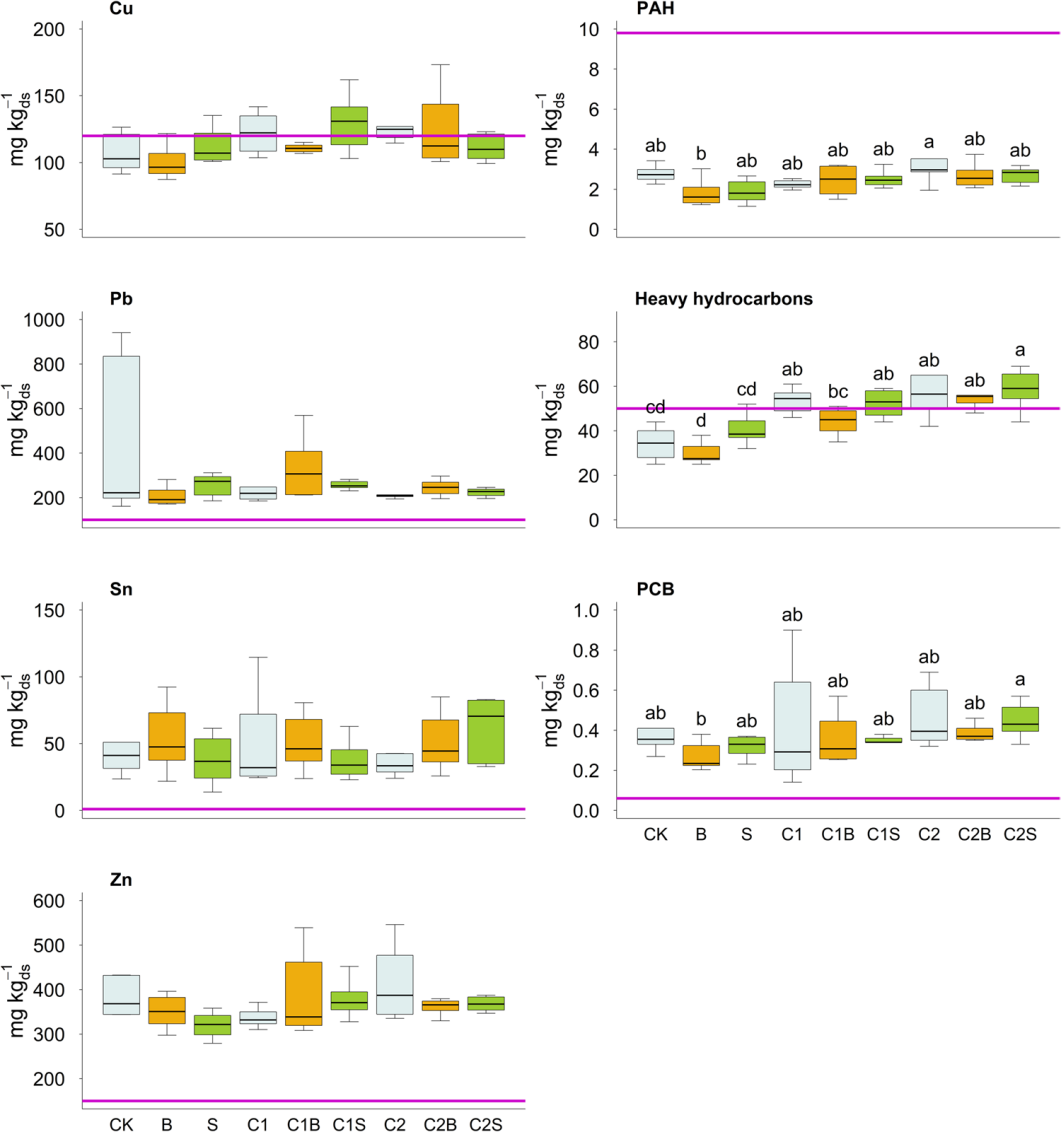
Polycyclic aromatic hydrocarbons (PAH), heavy hydrocarbons (> 12 carbon number), polychlorinated biphenyls (PCB), and Cu, Pb, Sn, and Zn contents in soil (expressed as mg per kg of dry soil — ds). Different lower-case letters indicate significant differences between the means (*p*-value < 0.05). Solid violet lines indicate Italian threshold limits for residential areas while CK = no plant, no compost, B = Brassica, S = Sorghum, C1 = compost 1, C1B/C1S = Brassica/Sorghum with compost 1, C2 = compost 2, and C2B/C2S = Brassica/Sorghum with compost 2

The content of PTE and organic contaminants in soil pots at the end of the trial is reported in Fig. 2. Concerning PTE, no significant differences among treatments emerged. PAHs and heavy hydrocarbons content decreased significantly in the presence of *Brassica* compared to *Sorghum* or no plant (−27% and −22% in [B] and [C2B] for PAHs and −16, −12, and −9% in [B], [C1B], and [C2B] for heavy hydrocarbons), whereas no significant plant effect on PCB reduction was found. On the other hand, a general adverse effect of compost on organic contaminants dissipation was observed, particularly for heavy hydrocarbons. In most cases, independently from the plant type, organic contamination followed the trend [C2] > [C1] > [CK].

### Trace element uptake

**Fig. 3:**
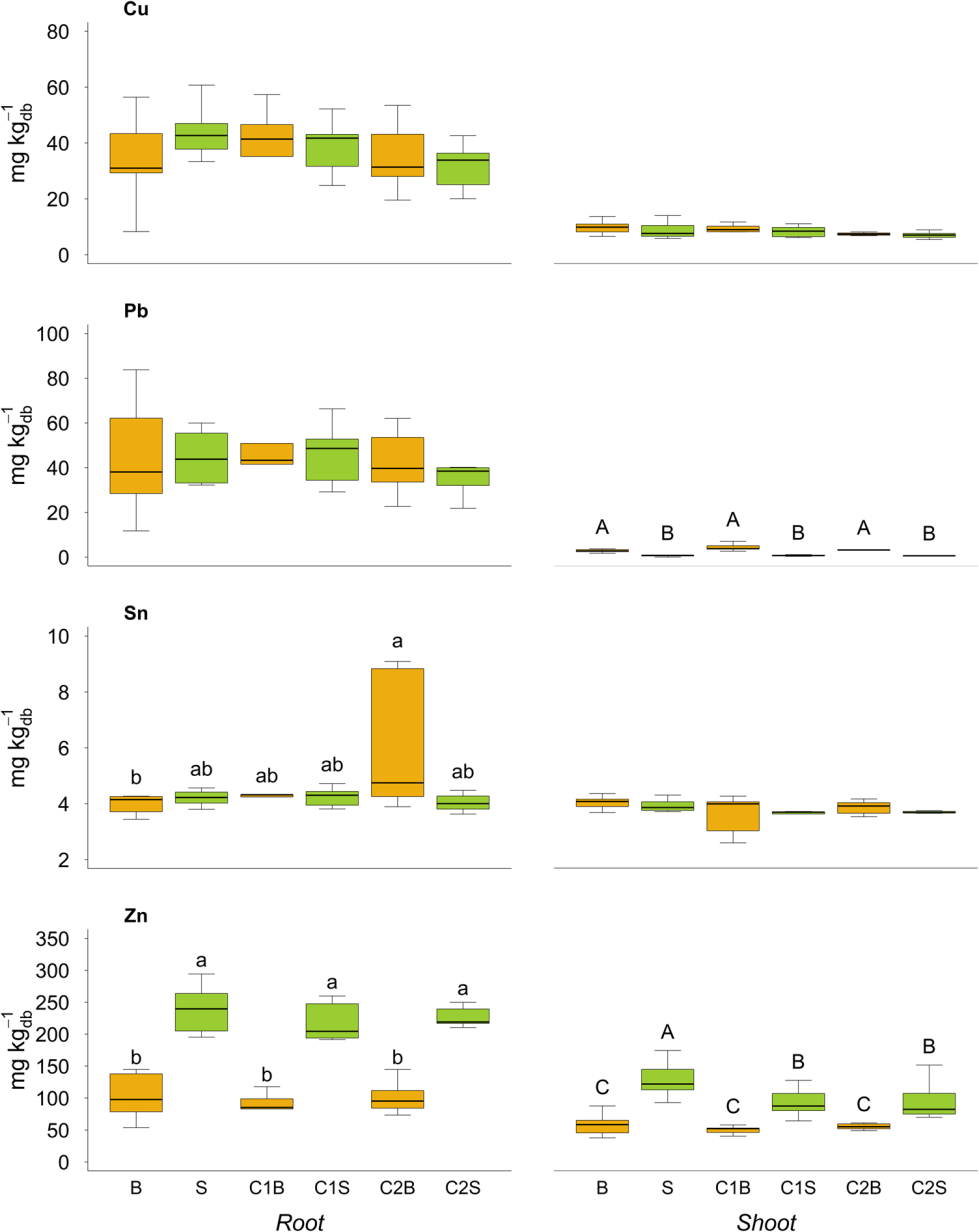
Content of Cu, Pb, Sn, and Zn in roots and shoots at the end of the trial (expressed as mg per kg of dry biomass). Different lower-case and upper-case letters indicate significant differences (*p*-value < 0.05) within the roots and the shoot, respectively. B = Brassica, S = Sorghum, C1B/C1S = Brassica/Sorghum with compost 1, and C2B/C2S = Brassica/Sorghum with compost 2

The total concentration of Pb and Sn, as well as the micronutrients Cu and Zn, was determined in roots and shoots of *Brassica* and *Sorghum* (Fig. 3). As a global average, plant roots and shoots accumulated 37.19 ± 11.49 and 8.40 ± 2.10 mg of Cu per kg_db_^−1^, respectively, with no significant differences among the experimental conditions. Similarly, roots accumulated 46.18 ± 22.39 mg kg_db_^−1^ of Pb, with no relevant differences. However, *Brassica* accumulated 80% more Pb in shoots when compared to *Sorghum*, but no compost effect was observed. Compost addition (C2 particularly) had a remarkable effect on Sn accumulation in *Brassica* roots [C2B], and in general, Sn concentrations in roots and shoots were similar (plant translocation factor ≈1). Zn content in *Brassica* roots was significantly lower (57%) with respect to *Sorghum*, but the shoots of *Sorghum* in the absence of compost [S] had the highest Zn concentration (128.3 ± 25.4 mg kg_db_^−1^). The Zn translocated in shoots of both plant species was about half as high as the respective root concentration.

### Soil chemical and biochemical indicators

**Fig. 4:**
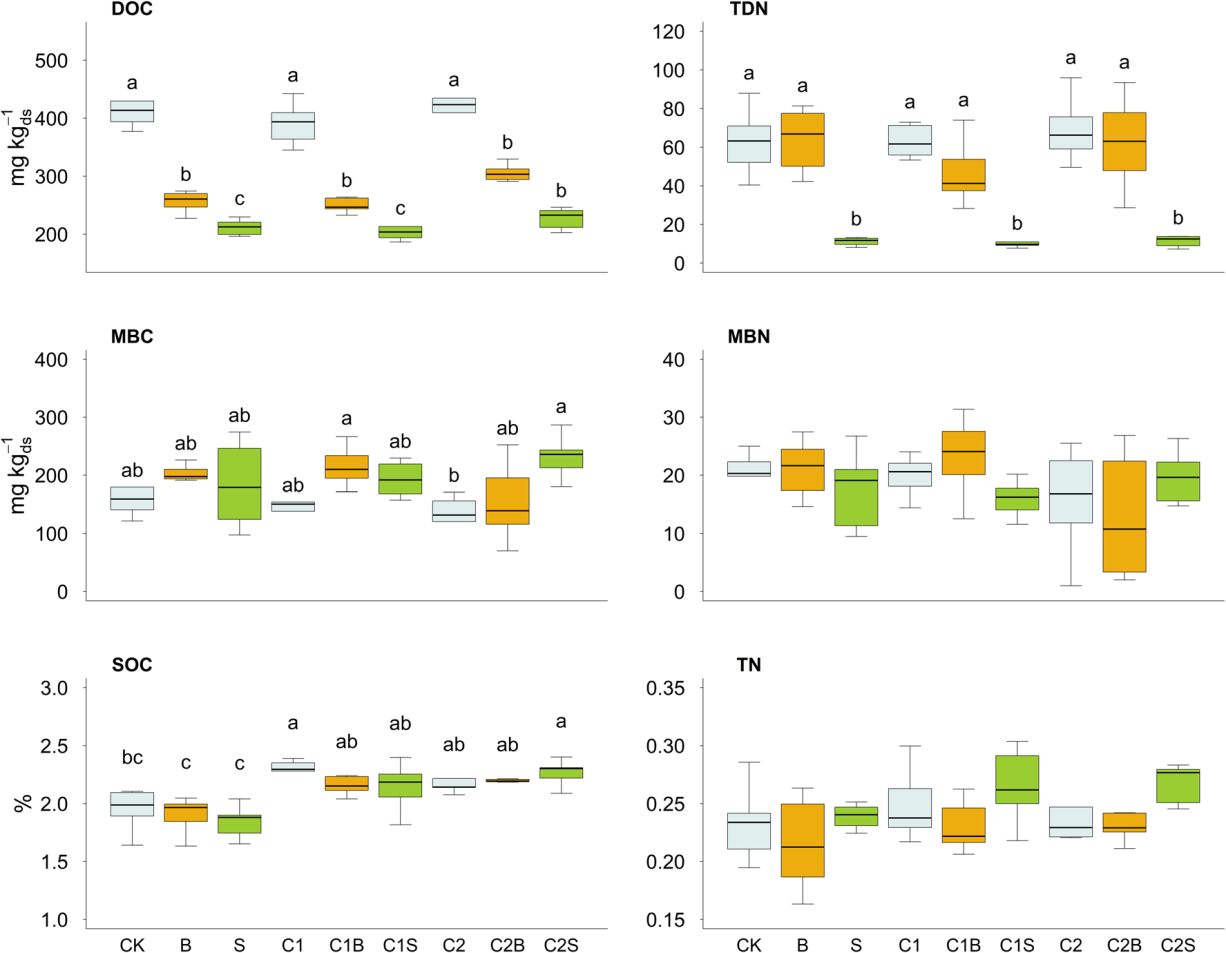
Content of soil dissolved organic carbon (DOC), microbial biomass carbon (MBC), total soil organic carbon (SOC), total dissolved nitrogen (TDN), microbial biomass nitrogen (MBN), and total nitrogen (TN). Different lower-case letters indicate significant differences (*p*-value < 0.05). CK = no plant, no compost, B = Brassica, S = Sorghum, C1 = compost 1, C1B/C1S = Brassica/Sorghum with compost 1, C2 = compost 2, and C2B/C2S = Brassica/Sorghum with compost 2

Soil carbon and nitrogen pools are reported in Fig. 4. DOC content in soil significantly decreased in the presence of both *Brassica* and *Sorghum* (−35 and −46%, respectively, *p* < 0.01), when compared to treatments without plant. In general, the MBC content turned out to be lower in absence of the plant, whereas higher values were measured for [C1B] and [C2S] (+23 and +26% compared to the average value of the other treatments). Higher SOC content was measured in correspondence of compost addition (an average value of 2.21% of SOC for [C1], [C1S], [C1B], [C2], [C2S], [C2B]), compared with treatments without compost [CK] [B] and [S] (with average value of 1.91 %) with only minor differences due to plants. TDN content showed significantly lower values in presence of *Sorghum* (-78%) independently from the compost addition.

Potential and specific soil enzymatic activities and the soil biochemical indexes are reported in Table 2. Potential β-glu activity showed higher values (+16% on average) with *Brassica* independently from compost addition, while specific β-Glu activity resulted to be higher with [C2] and [C2B] (+37% on average). On the contrary, both potential and specific Dehy activity indicated no differences among treatments. Regarding the biochemical indexes, the microbial quotient (qmic) resulted mainly affected by plants with lower values in the absence of plants (mean value of 7.25 mg_MBC_ g_SOC_^−1^), while minor differences occurred between *Brassica* and *Sorghum* (qmic increased by 21 and 26%, respectively, with *Brassica* and *Sorghum* at any compost level). A similar trend could be observed also for the metabolic index (MI) with lower values in absence of plant (0.278 mg_INTF_ mg_DOC_^−1^ h^−1^ on average) and increasing values in both *Brassica* and *Sorghum* (+42 and +53%, respectively).

**Table 2:** Marginal means of potential soil β-glucosidase activity (β-Glu, μg_pN_ g_ds_^−1^ h^−1^), potential dehydrogenase activity (Dehy, μg_INTF_ g_ds_^−1^ h^−1^), specific β-glucosidase activity (β-Glu_MBC_, μg_pN_ mg_MBC_^−1^ h^−1^), specific dehydrogenase activity (Dehy_MBC_, mg_INTF_ mg_MBC_^−1^ h^−1^), microbial quotient (qmic, mg_MBC_ g_SOC_^−1^), and metabolic index (MI, mg_INTF_ mg_DOC_^−1^ h^−1^). CK = no plant, no compost, B = brassica, S = sorghum, C1 = compost 1, C1B/C1S = brassica/sorghum with compost 1, C2 = compost 2, and C2B/C2S = brassica/sorghum with compost 2

Treatments	β-Glu	Dehy	β-Glu_MBC_	Dehy_MB_	MI	qmic
CK	0.732c	109	4.59ab	0.680	0.260d	8.66ab
B	0.972a	120	4.91ab	0.607	0.467bc	10.6a
S	0.873abc	124	5.41ab	0.785	0.601a	10.1ab
C1	0.853abc	112	5.90ab	0.771	0.289d	6.59b
C1B	0.994a	137	4.71ab	0.644	0.536abc	9.84ab
C1S	0.788bc	124	4.08b	0.605	0.607a	9.44ab
C2	0.911ab	122	7.70a	1.038	0.285d	6.51b
C2B	0.973a	130	7.45a	0.986	0.437c	7.06ab
C2S	0.809bc	142	3.77b	0.670	0.552ab	9.73ab
*p-value*	*0.006*	*0.276*	*0.007*	*0.199*	*0.016*	*0.014*

Lowercase letters following marginal mean values reflect Tukey’s HSD pairwise comparison at *p*-value < 0.05

### qPCR, NGS and microbial biodiversity indices

The presence of compost 2 in *Brassica* [C2B] significantly increased bacterial counts compared to *Brassica* units without compost [B] (Table 3). Instead, without compost, fungi were significantly higher with *Sorghum* [S] when compared to *Brassica* [B].

**Table 3:** Marginal means of soil qPCR counts of total bacteria (Log10 n. 16S rDNA copies g^−1^ dry soil) and fungi (Log10 n. 28S rDNA copies g^−1^ dry soil). CK = no plant, no compost, B = brassica, S = sorghum, C1 = compost 1, C1B/C1S = brassica/sorghum with compost 1, C2 = compost 2, and C2B/C2S = brassica/sorghum with compost 2

Treatments	Total bacteria	Total fungi
CK	9.58ab	7.10abc
B	9.34b	6.81c
S	9.66a	7.24a
C1	9.69a	7.01abc
C1B	9.49ab	6.84bc
C1S	9.53ab	7.09abc
C2	9.64a	6.88abc
C2B	9.72a	7.18ab
C2S	9.58ab	7.09abc
*p-value*	*0.001*	*0.001*

Lowercase letters following marginal mean values reflect Fisher's LDS pairwise comparison at *p*-value < 0.05

The samples obtained from pot tests were sequenced on an Illumina MiSeq platform. About 1.7 million raw reads were obtained from the sequencing. A sample from the experimental conditions [CK] at T0 and T1 and [S] and [C2] of T1 were discarded due to the very low number of reads. All analyzed samples were rarefied at 33,868 reads per sample. Final data on the relative abundance at phyla and family level are reported in Figures S1 - S2 and Tables S3 - S4.

### Microbial biodiversity indices

**Fig. 5:**
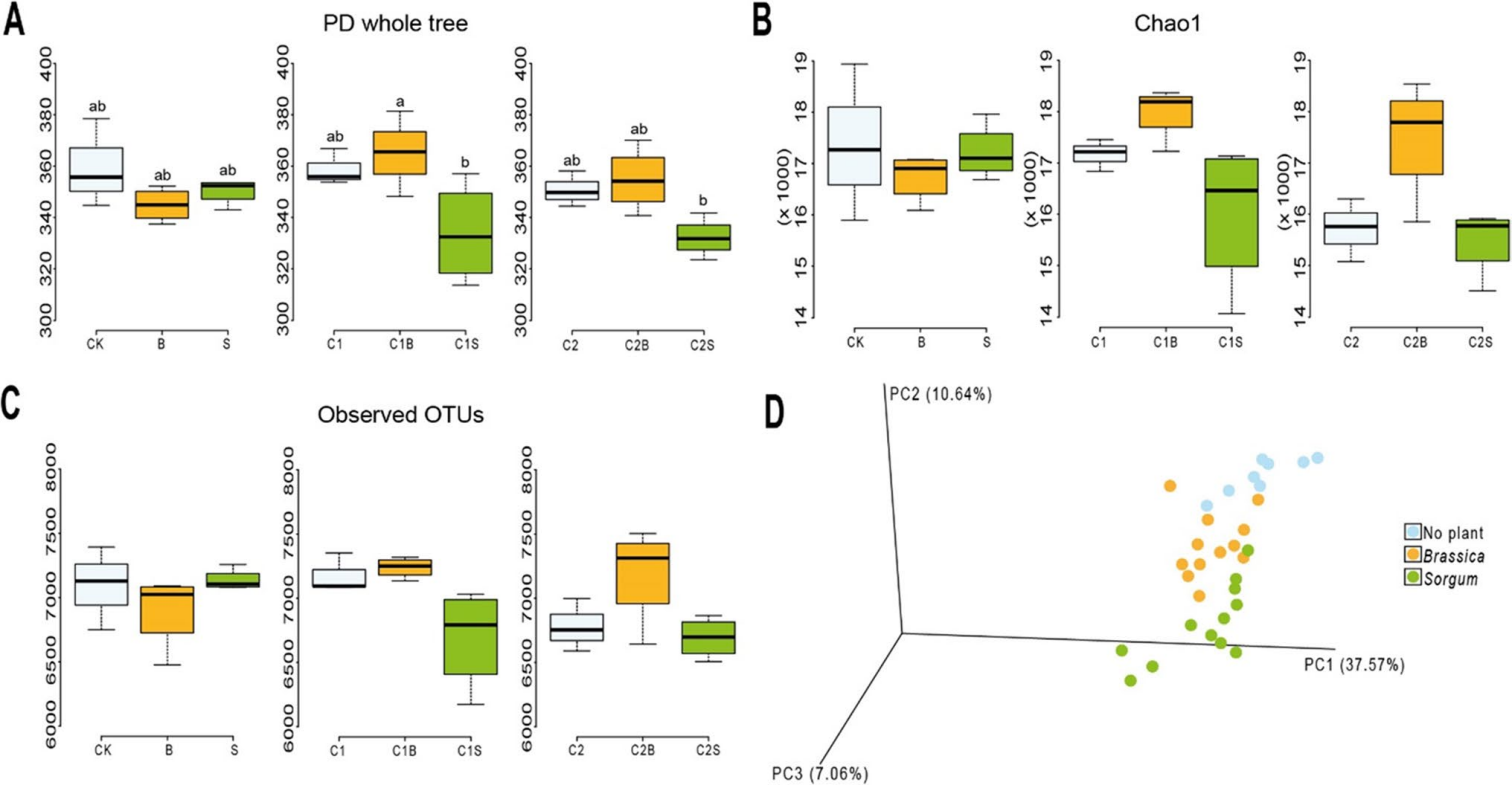
Biodiversity in soil microbiota within the experimental conditions expressed as α- and β-diversity. α-Diversity metrics: Chao1 (**A**), observed OTU (**B**), and PD whole tree (**C**). β-Diversity is represented as PCA on Weighted Unifrac distance (**D**). Different lower-case letters indicate significant differences (*p*-value < 0.05). CK = no plant, no compost, B = Brassica, S = Sorghum, C1 = compost 1, C1B/C1S = Brassica/Sorghum with compost 1, C2 = compost 2, and C2B/C2S = Brassica/Sorghum with compost 2

The biodiversity of the soil microbiota was evaluated through α- and β-diversity indexes T0 vs T1 (Fig. S3) and at T1 (Fig. 5). At T0, both α-diversity (Chao1, observed_OTU, and PD_whole_tree) and β-diversity (weighted and unweighted unifrac) metrics did not show significant differences among the three initial experimental conditions. At T1, α-diversity metrics showed a reduction in all experimental conditions with *Sorghum* and both composts, whereas the presence of *Brassica* and the two composts showed an opposite trend (Fig. 5C). Particularly, in the PD_whole_tree and Chao1 analysis, the divergence between the microbial population trends in [C1B-C2B] and [C1S-C2S] was significant when comparing [C1S] vs [C1B] and [C2S] vs [C2B] (Fig. 5A and B; *p*-value < 0.05). Observed_OTU index did not show any significant comparison among experimental conditions (Fig. 5C). In β-diversity, the presence/absence and the type of plant experimental conditions clustered separately in the PCA (Fig. 5D). Comparing data of α-diversity and β-diversity at T0 *vs* T1 also did not evidence any statistical difference.

### Comparison of the microbial profile among the different experimental conditions

**Fig. 6:**
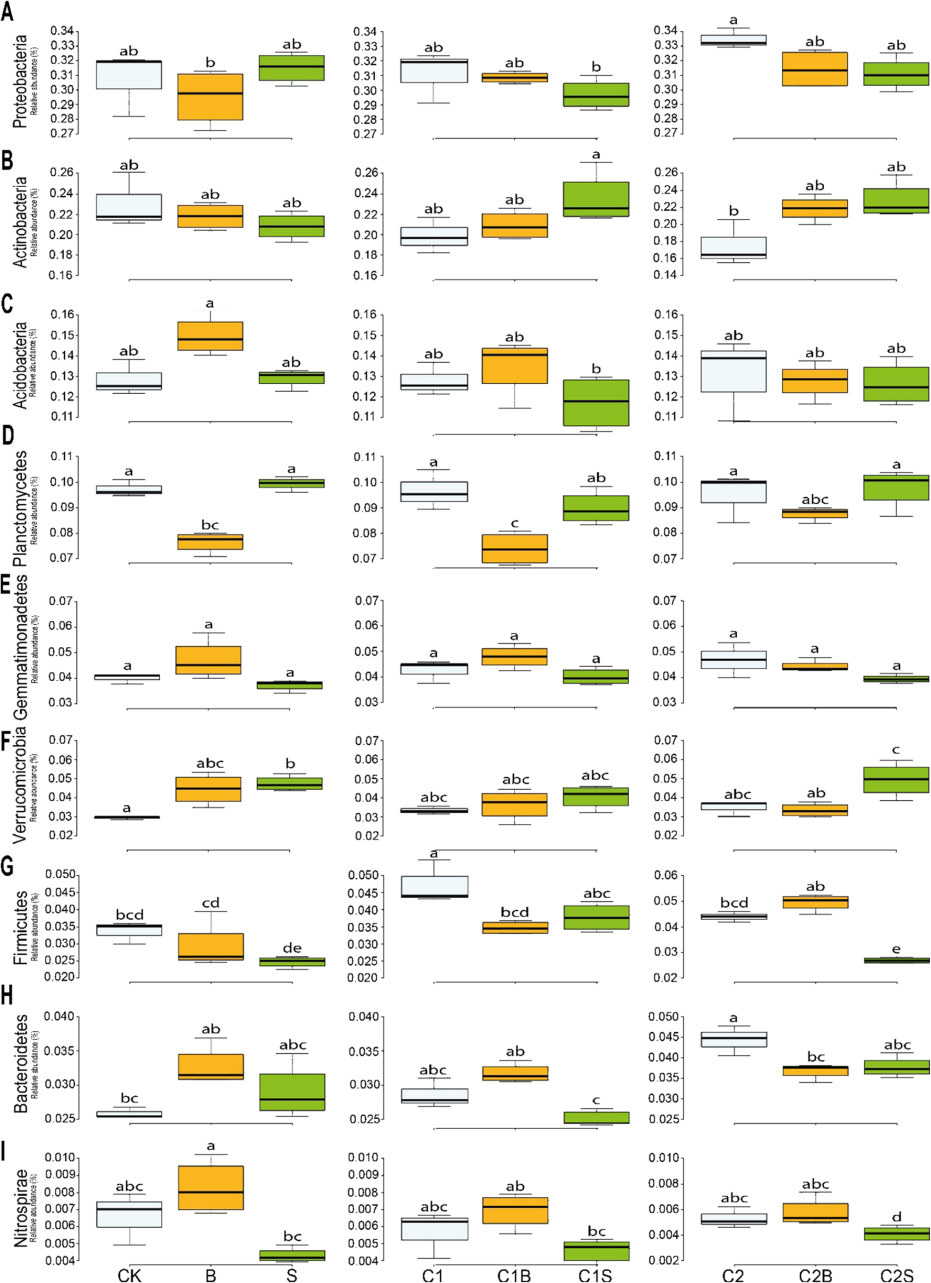
Relative abundances of bacterial phyla in soil within the experimental conditions. Different lower-case letters indicate significant differences (*p*-value < 0.05). CK = no plant, no compost, B = Brassica, S = Sorghum, C1 = compost 1, C1B/C1S = Brassica/Sorghum with compost 1, C2 = compost 2, and C2B/C2S = Brassica/Sorghum with compost 2

Nine major microbial phyla were detected in the soil samples (Fig. 6): Proteobacteria (average 30.98%; Fig. 5A), Actinobacteria (21.44%; Fig. 6B), Acidobacteria (13.03%, Fig. 6C), Planctomycetes (8.99%, Fig. 6D), Gemmatimonadetes (4.28%; Fig. 6E), Verrucomicrobia (3.94%), Firmicutes (3.25%), Bacteroidetes (2.88%), and Nitrospirae (0.57%), that altogether accounted for 90.03% of the total microbes present in the analyzed soil samples. For Planctomycetes, only the comparisons [CK] vs [B] and [C1] vs [C1B] resulted significant (*p*-value < 0.05) (Fig. 6D). Firmicutes showed significant pairwise comparison: for [C1], an increase of 1.3% was observed upon compost application with and without *Sorghum* (Fig. 6G). Concerning [C2], a significant increase was observed in relation to the crop presence and species (3.38% of [C2] vs 6.73% of [C2S]; 2.91% of [B] to 3.95% of [C2B]). The type of compost also affected Firmicutes proliferation registering an increase of this Phylum in *Sorghum* samples from 3.77% [C1S] to 6.73% [C2S] (*p*-value < 0.05). Firmicutes showed a 1.29% decrease comparing [C1] vs [C1B]. Verrucomicrobia significantly increased in the presence of *Sorghum* from 2.23% in [CK] to 4.12% in [S] and 4.32% in [C2S], and when [C2B] was compared to [C2S] (*p*-value < 0.05; Fig. 6F). Finally, Bacteroidetes significantly increased with [C2] and showed the lowest values within [CK] and [C1S] experimental conditions (Fig. 6H).

**Fig. 7:**
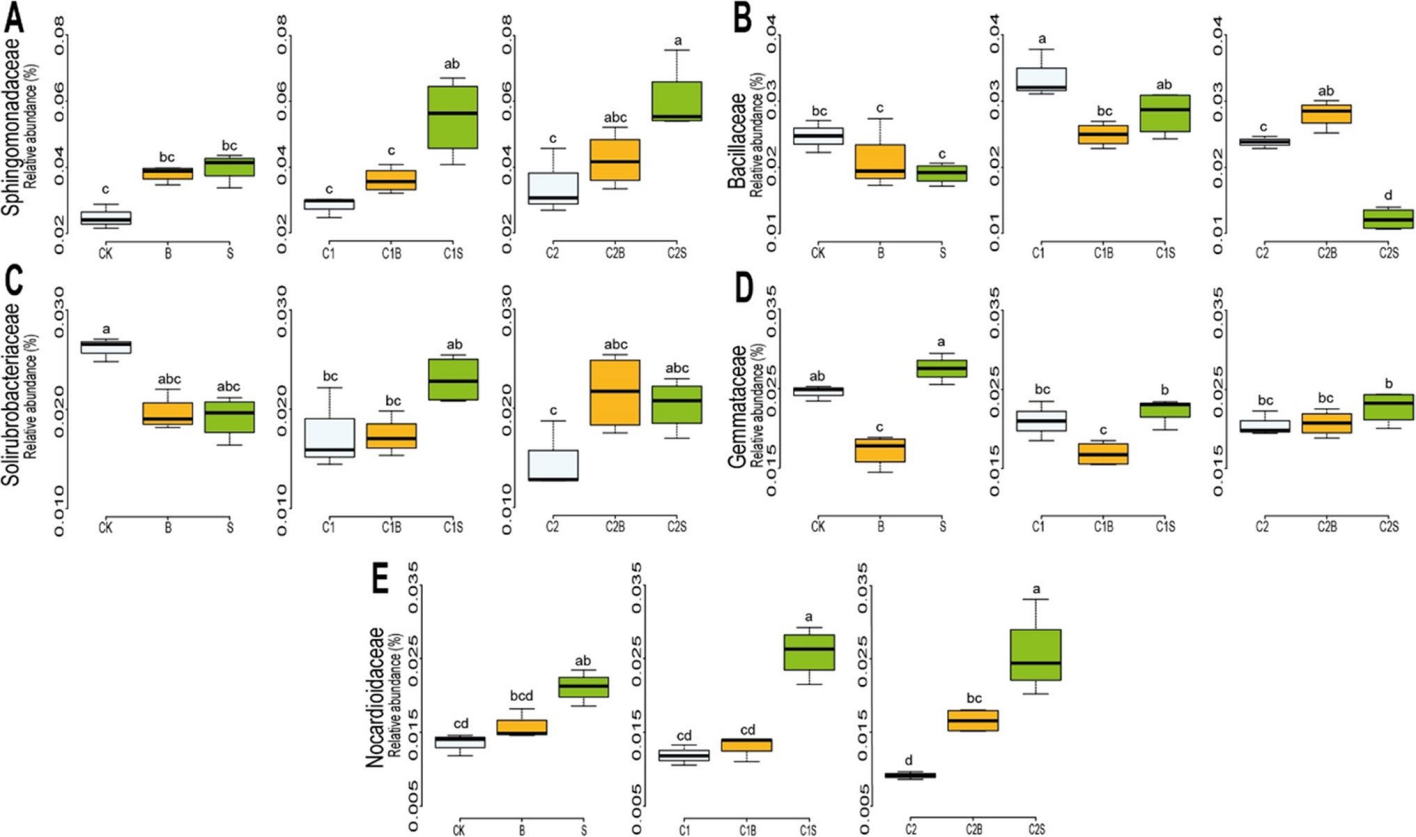
Relative abundances of bacterial families in soil with more variability within the experimental conditions. Different lower-case letters indicate significant differences (p-value < 0.05). CK = no plant, no compost, B = Brassica, S = Sorghum, C1 = compost 1, C1B/C1S = Brassica/Sorghum with compost 1, C2 = compost 2, and C2B/C2S = Brassica/Sorghum with compost 2

The microbial families showing relevant variations among experimental conditions are reported in Fig. 7. Among these families, the relative abundance of Sphingomonadaceae (Fig. 7A) increased in the presence of both *Brassica* and *Sorghum,* although only *Sorghum* was significant when compared with the other experimental conditions (*p*-value < 0.01). [C1] and [B] also increased the relative abundance of Bacillaceae, where [C1] and [C2B] showed a significant increase when compared to [CK] and [B], respectively (*p*-value < 0.01). On the contrary, [C2] alone or combined with *Sorghum* significantly reduced the relative abundance of Bacillaceae when compared to [C1] and [C1S] (*p*-value <0.01). Solirubrobacteriaceae relative abundance decreased significantly in [C1] and [C2] when compared to [CK] (*p*-value <0.01), whereas both *Sorghum* and *Brassica* did not impact this microbial group (Fig. 7C). Gemmataceae relative abundance resulted significantly lower in the presence of *Brassica* [B] when compared to [CK] and [S] (*p*-value < 0.01; Fig. 7D). Also, the presence of C1 combined with *Brassica* significantly lowered Gemmataceae when compared to [C1S] (*p*-value < 0.01) and to [C1] (*p*-value = 0.005). Instead, compost 2 stabilized Gemmataceae and no differences among [C2], [C2S], and [C2B] experimental conditions were highlighted. The relative abundance of Nocardioidaceae significantly increased with *Sorghum* (*p*-value < 0.01; Fig. 7E)*.*

### Pearson’s correlation between soil microbial groups, soil pollutants, and plant biomass

**Fig. 8:**
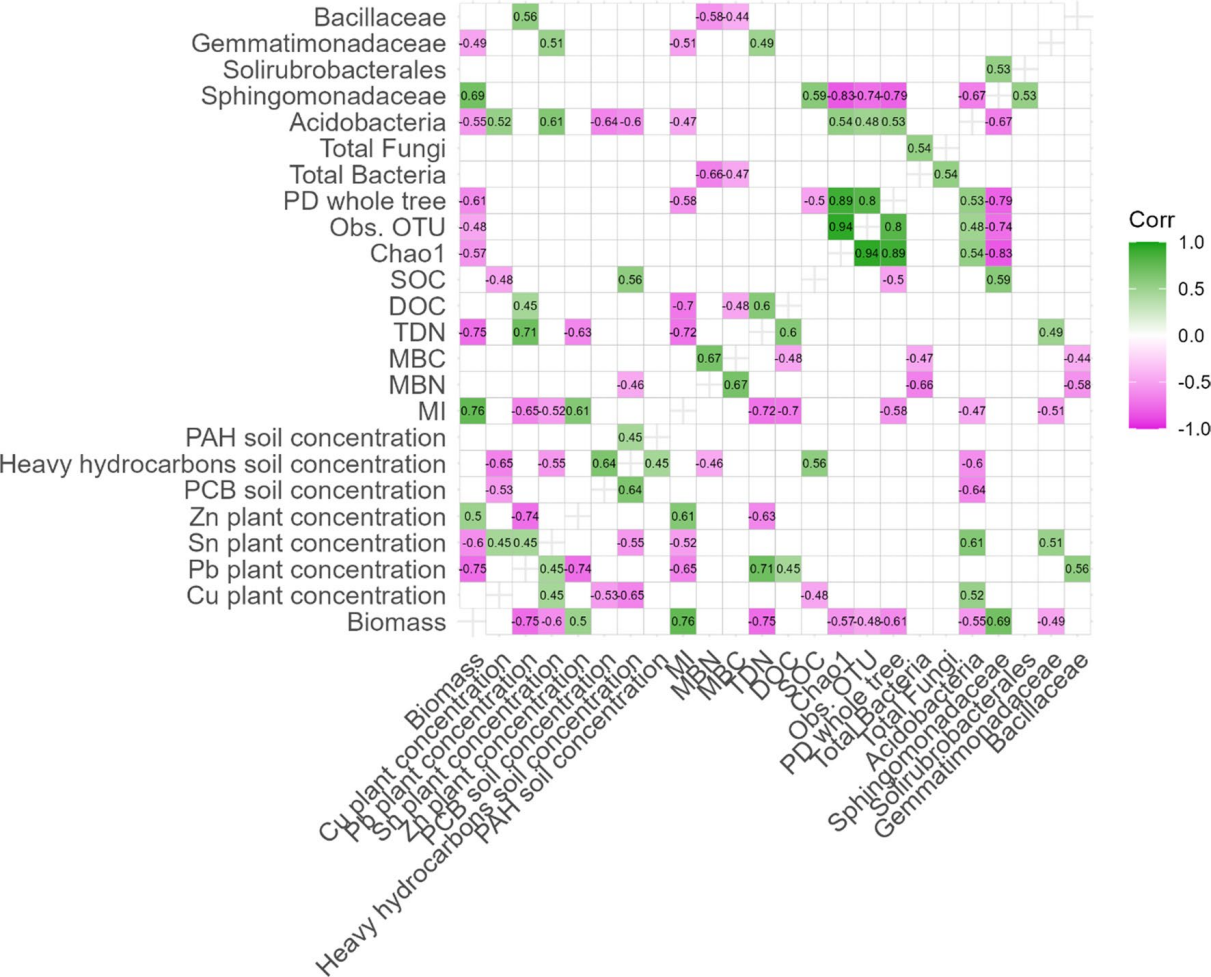
Pearson’s correlation coefficients between main soil, plants and microbiological parameters: plant Cu content (mg kg_db_^−1^), plant Pb content (mg kg_db_^−1^), plant Sn content (mg kg_db_^−1^), plant Zn content (mg kg_db_^−1^), plant biomass (plant biomass, g), α-diversity metrics (Chao1, Obs. OTU, PD whole tree), total bacteria (Log10 n. 16S rDNA copies g_ds_^−1^), total fungi (Log10 n. 28S rDNA copies g_ds_^−1^), relative abundance of acidobacteria (Acidobacteria; subgroup 6), relative abundance of bacterial families (Sphingomonadaceae, Solirubrobacterales, Gemmatimonadaceae, Bacillaceae), dissolved organic carbon (DOC, mg kg_ds_^−1^), total soil organic carbon (SOC, %) total dissolved nitrogen (TDN, mg kg_ds_^−1^), microbial biomass carbon (MBC, mg kg_ds_^−1^), total dissolved nitrogen (MBN, mg kg_ds_^−1^), metabolic index (MI, mg_INTF_ mg_DOC_^−1^ h^−1^), polycyclic aromatic hydrocarbons (PAHs, mg kg_ds_^−1^), heavy hydrocarbons (C12), and polychlorinated biphenyls (PCB). Coefficients associated to insignificant *p*-values (>0.05) are not shown. Correlation coefficients were built using *N* = 22 observations

Considering the Pearson’s correlation coefficients between different PTE contents in shoots (Fig. 8), high positive correlations were observed between Cu and Sn (*r* = 0.44), and negative correlation between Cu and Pb (*r* = -0.74). Additionally, shoot biomass was positively correlated with Zn, and negatively correlated with Pb and Sn content in shoots (*r* = 0.50, −0.75, and −0.50, respectively). Considering the correlations between some of the most abundant bacterial families and soil biochemical properties or plant parameters, a negative correlation between PCB and observed Acidobacteria or Cu content in plant biomass was observed (*r* = −0.61 and −0.53, respectively), as well as the negative association between total bacteria counts and MBC or MBN (*r* = −0.48 and −0.62, respectively). Furthermore, Acidobacteria and Sphingomonadaceae showed an opposite trend: with plant biomass, heavy hydrocarbon content, and α-diversity indices, showing a selective effect of the pollutants towards the different microbial groups.

## Discussion

Phytoremediation is considered a promising tool for the decontamination of polluted soils; however, a better understanding of the mechanisms underlying soil pollutant accumulation and tolerance is envisaged (Yan et al. 2020). Typically, phytoremediation trials address only a few pollutants at a time and determine biochemical or microbiological parameters separately. In this work, we simultaneously measured the phytoremediation potential on a wide range of soil pollutants, clarifying the adsorption efficacy in combination with two different organic amendments. Despite the short-term experiment conducted, significant results were obtained for both inorganic and organic contaminants in terms of plant growth, soil biochemistry, and soil microbial community.

Concerning PTE, the positive correlations between Cu and Sn content in plants may be interpreted as the result of a proportional metal uptake mediated by non-specific symplastic transport mechanisms (Demidchik et al. 2002). Moreover, the positive correlation between Zn absorbed by the plants and plant biomass, as well as between plant biomass and Sphingomonadaceae, can be linked to the documented ability of members of this taxon to promote plant growth (Gatheru Waigi et al. 2017). These beneficial plant–microbe interactions, which are vital to plant survival in contaminated environments (Rajkumar et al. 2012), may be enhanced by the combination *Sorghum*-composts highlighting a potentially successful remediation strategy for polluted soil.

*Brassica* cultivation without compost slightly reduced the concentration of all organic contaminants (PAH, heavy hydrocarbons, and PCB). On the other hand, an adverse effect on contaminants degradation was observed upon compost addition both with and without plants (*Brassica* or *Sorghum*). In general, the addition of compost has been found to allow organic contaminant removal to be enhanced by improving nutrient contents as well as biomass content and activity of microorganisms (Lwin et al. 2018; Ventorino et al. 2019). Moreover, in a 6-month study of Baldantoni et al. (2017), the reduction of hydrocarbon content was higher in the presence of organic amendment (140 g of compost in 23 kg of soil) than in the untreated control (52% reduction in compost amended units compared to 46% reduction in control units). Differently, in our study, compost addition did not decrease the content of organic contaminants in the soil. This result could be supported by the following observations: (i) the two partially stabilized organic materials did not fit with the purpose of stimulating the soil decontamination; (ii) longer time was needed to observe a significant and positive effect of compost on organic contaminants degradation; (iii) the soil was already in a stabilized condition in terms of C content (soil without plant and without compost had a SOC content of 2% that is fairly high for Italian soils). On the other hand, the increase of some microbial families and related genera, such as Sphingomonadaceae and *Sphingomonas* in [C1S], as well as Nocardoidaceae and *Nocardoides* in *Sorghum* with both composts, is potentially a favorable condition for decontamination activity. Indeed, Sphingomonadaceae related members possess biodegradation abilities against dangerous organic compounds, such as PAH, dioxins, and chlorinated phenols (Colquhoun et al. 2012; Hesham et al. 2014; Matsumura et al. 2015; Onder Erguven and Demirci 2020). *Nocardoides* members are well-known aliphatic and aromatic hydrocarbon-degrading bacteria (Schippers et al. 2005), in particular in PAH, 2,4-dinitroanisole (DNAN) and total petroleum hydrocarbons (Karthikeyan and Spain 2016; Khudur et al. 2019; Lu et al. 2019). However, the impact of compost at the experiment start (T0) and at the end of the experiment (T1) seems moderate. On the contrary, the major microbial changes should be attributed to the plant cultivated within the contaminated soil.

In our study, both the composts induced an increase (+14%) in the SOC content compared to the control soil and regardless of the plant species that, nevertheless, appeared to be insufficient to enhance the PTE mobilization and plant uptake. However, this result could not be ascribed to the heavy metals content in the composts as the concentrations were significantly below the limits of the Italian law and the amount of compost added to the soil was low (1.2% w/w); moreover, no significant results were observed in PTE soil content at the end of the experimentation. On the contrary, a slight increase of DOC was observed with [C2] and it was significant only with *Sorghum* (+23%), while, as already observed in previous studies (Lwin et al. 2018; Masciandaro et al. 2013; Sciubba et al. 2015), the MBC content was increased by the compost-plant interaction ([C1B] and [C2S]). In our study, even if an increased MBC content was measured, a corresponding increase of the enzymatic activities of β-Glu and Dehy was not observed. Therefore, the addition of compost increased the microbial biomass but not its activity due to the low stability of the organic material added to the soil. However, the mixture of PTE and organic contaminants probably acted as an inhibitor for the enzyme activities measured as well (Bello et al. 2014; Tang et al. 2014). Moreover, even though microbial biomass varied due to the presence of plants and their stimulation of microbial growth in proximity of the plant roots, this interaction cannot be correlated with specific bacterial groups. In fact, different microbial components, such as Solirubrobacteriaceae, Bacillaceae, and Gemmataceae, showed a different response to the presence of compost. Overall, the combination [C1S] or [C2S] led to a reduction of bacterial biodiversity, which agrees with the higher specialization of the rhizosphere microbiota with respect to that of the bulk soil (Fierer 2017). An additional interesting aspect was that, in many cases, the two types of compost gave different results, indicating that the type of compost had a crucial importance not only for the remediation process but also for the maintenance and/or increase of soil quality and fertility (Sciubba et al. 2015).

No considerable variations of total bacteria and fungi were detected in the experimental conditions, apart from a reduction of both microbial communities only in the presence of *Brassica* without any compost. Based on this evidence, a deficiency of essential elements for the metabolism of bacteria and fungi can be supposed, although no decrease of SOC or TN was registered. However, the negative correlation of MBC and MBN with bacteria amount can be explained by the root selection of a specialized microbiota. Even though no significant associations were found between soil contaminants and specific microbial taxa, the Sphingomonadaceae plant growth-promoting traits have been confirmed, especially in the presence of *Sorghum.*

The use of the two composts and plants, as well as their combination, established complex interactions with the soil microbiota. To this extent, the significant reduction of Bacillaceae and *Bacillus* genus in the presence of *Sorghum* and [C2] is a clear evidence. Due to their ability to produce endospores, Bacillaceae are very resistant to adverse conditions. It is possible that, under the presence of an insufficiently stabilized compost associated with *Sorghum*, the increase of some other microbial groups may have been favored. In this context, Sphingomonadaceae and Nocardoidaceae are more easily adaptable to the experimental soil, owing to their high contaminant degradation ability, to the detriment of Bacillaceae members. *Sorghum*, regardless the type of compost used, determined a reduction of Nitrospirae, which is considered one of the keystone taxa in polluted soils, as it plays a role in maintaining the structure and function of ecological communities (Geng et al. 2020; Jiao et al. 2016; Yu et al. 2020). The detected reduction of Nitrospirae, which are nitrite-oxidizing bacteria that participate in the N cycle and are very common in soils (Lin et al. 2018; Zhu et al. 2019), agrees with *Sorghum* ability to release biological nitrification inhibitors (Li et al. 2021) and can be linked to the decrease of TDN in the same condition. Moreover, the significant reduction of Planctomycetes (and the family Pirellulaceae) and Gemmataceae in *Brassica* and a restoration by adding [C2] were highlighted. It is known that microbial growth on complex heteropolysaccharides and the oligo- and mono-saccharides produced from their hydrolysis originates major ecological niches for Planctomycetes in nature (Wang et al. 2015). Therefore, the presence of [C2] might have assured the availability of polysaccharides deriving from plants to be utilized by this bacterial community.

In this study, the experimentation setup (i.e., one vegetative cycle, “out of field” conditions, soil amendment with poorly stabilized composts) can be considered a first step to define the best potential decontamination ability of the two selected plant species and needs to be transferred to real-filed conditions for a deeper evaluation. However, in Italy, the complex national policy on the use of contaminated soils needs to be considered in launching experimental setups for decontaminating purpose. For this reason, studies taking into consideration not only the remediation *per se* but also the effects on soil biochemistry and microbiology are of utmost importance in delineating significant guidelines to better approach on-field phytoremediation processes in the long term.

## Conclusion

This study highlighted a contrasting effect of compost addition on the depollution ability of a polluted Italian soil. On one hand, adding compost to contaminated soil had a positive effect by slightly improving plant biomass, soil biochemical indicators, α-diversity, and the relative abundance of a few microbial groups, despite not always significantly. On the other hand, compost addition did not improve PTE uptake efficiency and slowed down the dissipation of organic contaminants. In our findings, compost had an adverse effect on organic pollutant degradation so, more generally, the best “recipe” for an efficient phytoremediation should be case based.

Ultimately, most of differences on observed variables were due to the choice of plant species. In general, *Sorghum* (i) showed higher biomass production, (ii) promoted higher microbial biomass carbon content, and (iii) favored specific microbial taxa (i.e., Sphingomonadaceae). Brassica showed better performance in the degradation of organic pollutants but, contrarily to what generally may be expected, did not show element uptake typical of an hyperaccumulator species.

In this light, when approaching soil remediation from both organic and inorganic contaminants, the choice of plant species and compost should be considered of fundamental importance to reach suitable results within phytoremediation.

### Supplementary information

ESM 1 (PDF 557 KB)

## Data Availability

The sequence data have been submitted to the NCBI repository Sequence Read Archive (SRA) databases under accession numbers SAMN26504741 to SAMN26504773, with BioProject number PRJNA813521. Supplemental information and data, including excel files of elaborated NGS data categorized at phyla, family, and genera levels, are available on reasonable request from the corresponding author.
