# Supplementary material for: Pot experimental trial for assessing the role of different composts on decontamination and reclamation of a polluted soil from an illegal dump site in Southern Italy using Brassica juncea and Sorghum bicolor
Source: Environ Sci Pollut Res Int. 2023 Dec 8;31(2):2640–56. doi: 10.1007/s11356-023-31256-3 (PMC10791941; doi:10.1007/s11356-023-31256-3)
Supplement: Supplementary file 1 — ESM 1 (PDF 557 KB) [file 11356_2023_31256_MOESM1_ESM.pdf]

SUPPORTING MATERIAL FOR:

Pot experimental trial for assessing the role of different composts on decontamination and reclamation of a polluted soil from an illegal dump site in Southern Italy using *Brassica juncea* and *Sorghum bicolor*.

Martina Mazzon, Nicole Bozzi Cionci, Enrico Buscaroli*, Daniele Alberoni, Loredana Baffoni, Diana Di Gioia, Claudio Marzadori, Lorenzo Barbanti, Attilio Toscano, Ilaria Braschi

Department of Agricultural and Food Sciences - Alma Mater Studiorum University of Bologna, Bologna (BO), Italy

*Corresponding author: Enrico Buscaroli, enrico.buscaroli2@unibo.it

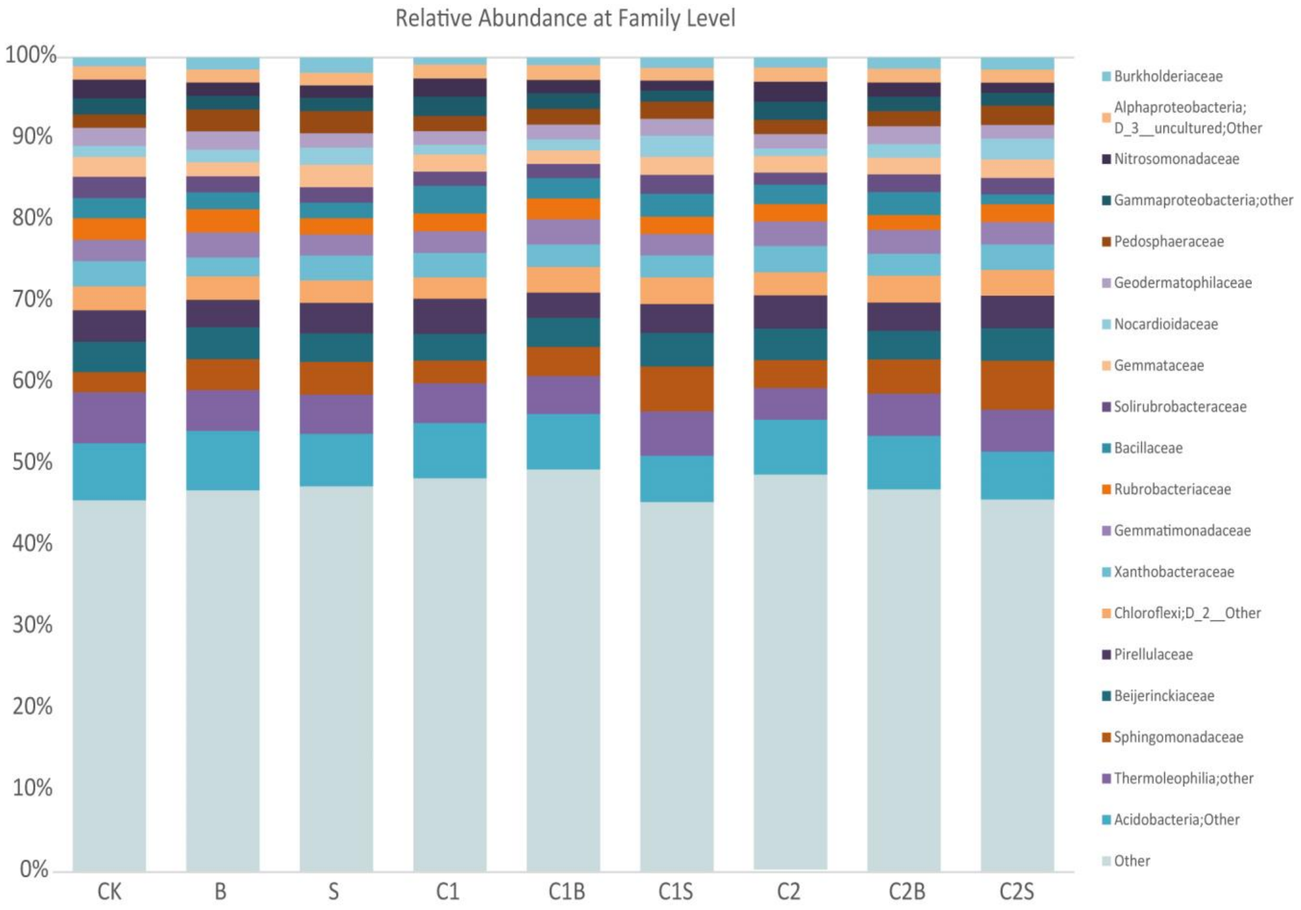

Fig. S1 Bar chart reporting NGS relative abundance of the major cumulated microbial Phyla per experimental condition at T1: CK = no plant, no compost, B = Brassica, S = Sorghum, C1 = compost 1, C1B/C1S = Brassica/Sorghum with compost 1, C2 = compost 2, C2B/C2S = Brassica/Sorghum with compost 2.

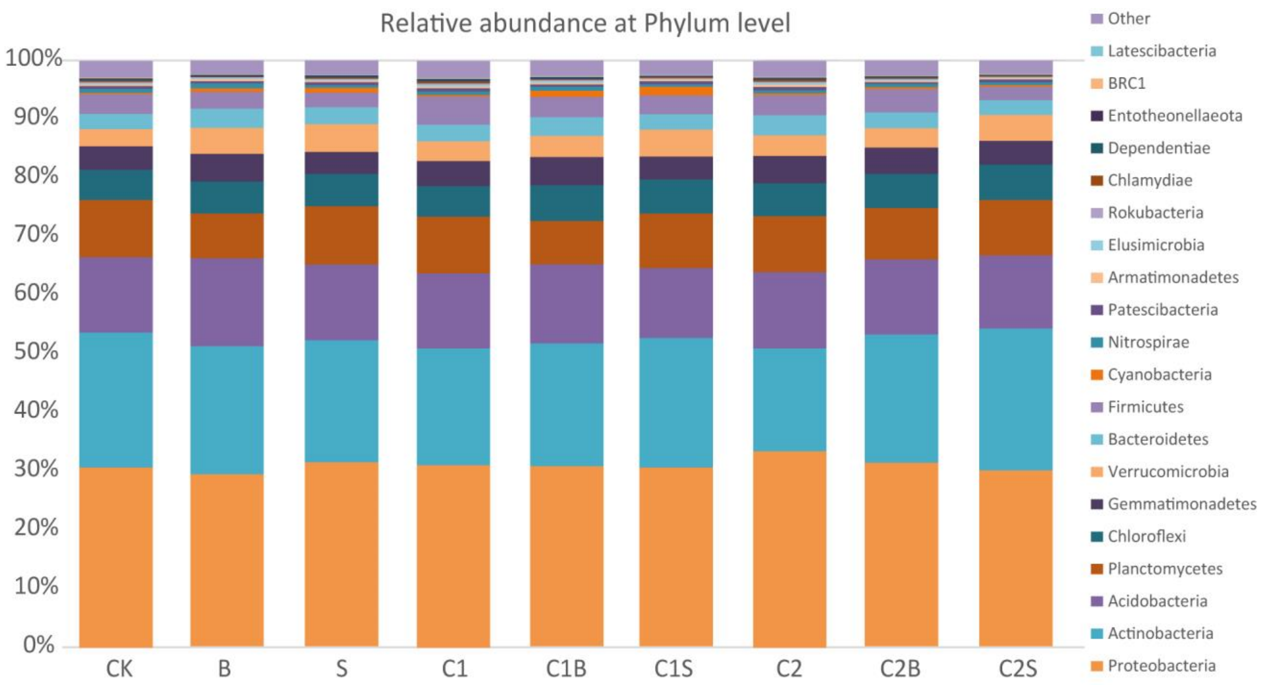

Fig. S2 Bar chart reporting NGS relative abundance of the major cumulated microbial Families per experimental condition at T1: CK = no plant, no compost, B = Brassica, S = Sorghum, C1 = compost 1, C1B/C1S = Brassica/Sorghum with compost 1, C2 = compost 2, C2B/C2S = Brassica/Sorghum with compost 2.

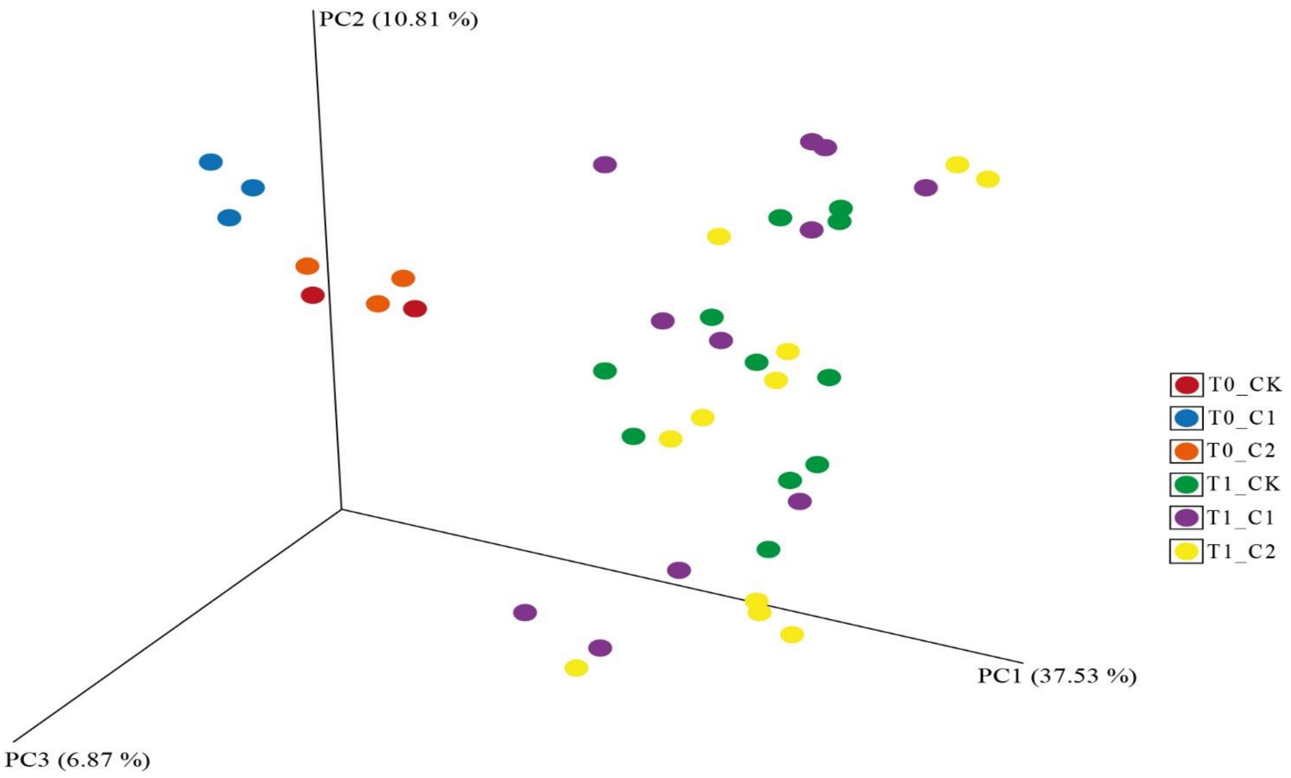

Fig. S3 PCA of β-Diversity (Weighted Unifrac Distance) showing the impact of the different composts [C1] and [C2] on soil at T0 and T1. T0= experiment beginning; T1= experiment end; CK = no compost; C1 = compost 1; C2 = compost 2.

Tab. S1 Physical and chemical characterisation of Compost 1 and 2 in compliance with Italian D. Lgs. 75/2010. Units of measure in square brackets. dm: dry matter.

**Parameter**	**Compost 1**	**Compost 2**
Humidity [%]	34	33
pH	6.86	6.44
Conductivity [mS cm^{-1}]	7.24	6.01
Germination Index	67	0
Organic carbon [%]	32.5	36.4
Total nitrogen [%]	2.31	1.89
C/N ratio	14	19
Al [mg kg_{dm}^{-1}]	12.7	3.11
Ca [mg kg_{dm}^{-1}]	163	3459
Co [mg kg_{dm}^{-1}]	0.01	0.01
Cr [mg kg_{dm}^{-1}]	0.08	0.11
Cu [mg kg_{dm}^{-1}]	1.26	0.94
Fe [mg kg_{dm}^{-1}]	15.3	7.61
Mg [mg kg_{dm}^{-1}]	48.9	584
P [mg kg_{dm}^{-1}]	85.9	32.8
Pb [mg kg_{dm}^{-1}]	0	0
S [mg kg_{dm}^{-1}]	257	621
Si [mg kg_{dm}^{-1}]	10.1	3.42
Sn [mg kg_{dm}^{-1}]	0.04	0.05
Sr [mg kg_{dm}^{-1}]	1.02	15.4
Zn [mg kg_{dm}^{-1}]	1.2	1.46

Tab. S2 Primers used in qPCR and NGS analysis.

Target	Primer sequence (5’-3’)	Amplicon (bp)	Annealing temperature (°C)	References
**Total bacteria**				
Eub338F<br>Eub518R	ACTCCTACGGGAGGCAGCAG<br>ATTACCGCGGCTGCTGG	200	60	Guo et al., 2008
**Total fungi**				
Yeast-F<br>Yeast-R	GAGTCGAGTTGTTTGGGAATGC<br>TCTCTTTCCAAAGTTCTTTTCATCTTT	124	60	Hierro et al., 2006
**V3-V4 region of the 16S rRNA**				
Pro341F<br>Pro805R	CCTACGGGNBGCASCAG<br>GACTACNVGGGTATCAATCC	460	60	Takahashi et al., 2014

Tab. S3 Relative abundance (%) at phylum level per microbial taxa and sample at T1. CK = no plant, no compost, B = Brassica, S = Sorghum, C1 = compost 1, C1B/C1S = Brassica/Sorghum with compost 1, C2 = compost 2, C2B/C2S = Brassica/Sorghum with compost 2.

Sample N.	Expeimental condition	Sampling time	Firmicutes	Proteobacteria	Actinobacteria	Acidobacteria	Planctomycetes	Chloroflexi	Gemmatimonadetes	Verrucomicrobia	Bacteroidetes	Cyanobacteria	Nitrospirae	Patescibacteria	Other
S	CK	T0	0.02	0.31	0.31	0.10	0.06	0.05	0.03	0.03	0.06	0.00	0.00	0.01	0.03
S	CK	T0	0.02	0.33	0.29	0.10	0.07	0.05	0.04	0.02	0.05	0.00	0.00	0.01	0.03
SC1	C1	T0	0.04	0.31	0.29	0.09	0.05	0.05	0.03	0.03	0.07	0.00	0.00	0.00	0.03
SC1	C1	T0	0.04	0.32	0.28	0.09	0.05	0.04	0.03	0.03	0.09	0.00	0.00	0.00	0.03
SC1	C1	T0	0.04	0.33	0.26	0.08	0.05	0.04	0.03	0.03	0.09	0.00	0.00	0.00	0.03
SC2	C2	T0	0.02	0.33	0.27	0.11	0.06	0.04	0.03	0.03	0.06	0.00	0.00	0.01	0.03
SC2	C2	T0	0.02	0.34	0.27	0.10	0.06	0.04	0.03	0.03	0.07	0.00	0.00	0.01	0.03
SC2	C2	T0	0.02	0.32	0.28	0.11	0.05	0.04	0.03	0.03	0.08	0.00	0.00	0.01	0.03
S.65	CK	T1	0.04	0.32	0.22	0.14	0.10	0.04	0.04	0.03	0.03	0.00	0.01	0.00	0.02
S.67	CK	T1	0.03	0.28	0.26	0.13	0.09	0.06	0.04	0.03	0.03	0.00	0.00	0.00	0.03
S.70	CK	T1	0.04	0.32	0.21	0.12	0.10	0.05	0.04	0.03	0.03	0.00	0.01	0.01	0.03
S.73	C1	T1	0.06	0.32	0.20	0.12	0.09	0.06	0.04	0.03	0.03	0.00	0.01	0.01	0.03
S.75	C1	T1	0.04	0.29	0.22	0.13	0.10	0.06	0.05	0.04	0.03	0.00	0.01	0.01	0.03
S.76	C1	T1	0.04	0.32	0.18	0.14	0.10	0.05	0.04	0.03	0.03	0.01	0.00	0.01	0.03
S.78	C2	T1	0.03	0.33	0.16	0.14	0.10	0.05	0.04	0.04	0.04	0.00	0.01	0.01	0.03
S.80	C2	T1	0.04	0.33	0.16	0.15	0.10	0.05	0.05	0.04	0.03	0.00	0.01	0.01	0.03
S.82	C2	T1	0.03	0.34	0.21	0.11	0.08	0.07	0.05	0.03	0.03	0.00	0.00	0.00	0.02
S.9	B	T1	0.04	0.29	0.23	0.15	0.07	0.06	0.04	0.04	0.03	0.00	0.01	0.00	0.02
S.11	B	T1	0.03	0.31	0.21	0.15	0.08	0.06	0.05	0.03	0.04	0.00	0.01	0.00	0.02
S.12	B	T1	0.03	0.27	0.23	0.14	0.08	0.06	0.06	0.05	0.03	0.01	0.01	0.00	0.02
S.13	B	T1	0.02	0.31	0.20	0.16	0.08	0.05	0.04	0.05	0.03	0.00	0.01	0.00	0.02
S.21	C1B	T1	0.04	0.31	0.22	0.14	0.07	0.06	0.04	0.04	0.03	0.01	0.01	0.00	0.03
S.22	C1B	T1	0.03	0.31	0.20	0.14	0.08	0.06	0.05	0.04	0.03	0.01	0.01	0.00	0.02
S.23	C1B	T1	0.03	0.30	0.20	0.15	0.08	0.06	0.05	0.04	0.03	0.01	0.01	0.00	0.03
S.24	C1B	T1	0.04	0.31	0.23	0.11	0.07	0.07	0.05	0.03	0.03	0.01	0.01	0.00	0.03
S.29	C2B	T1	0.03	0.30	0.24	0.13	0.09	0.06	0.04	0.03	0.03	0.00	0.01	0.00	0.03
S.30	C2B	T1	0.04	0.30	0.22	0.14	0.09	0.06	0.04	0.04	0.02	0.00	0.01	0.00	0.03
S.31	C2B	T1	0.04	0.33	0.20	0.13	0.09	0.06	0.04	0.03	0.03	0.00	0.01	0.00	0.03
S.32	C2B	T1	0.04	0.32	0.22	0.12	0.08	0.06	0.05	0.03	0.03	0.00	0.00	0.00	0.02
S.33	S	T1	0.02	0.30	0.22	0.13	0.10	0.06	0.04	0.04	0.03	0.00	0.00	0.00	0.02
S.35	S	T1	0.03	0.31	0.19	0.12	0.10	0.06	0.03	0.05	0.03	0.02	0.00	0.01	0.03
S.36	S	T1	0.03	0.32	0.20	0.13	0.10	0.05	0.04	0.05	0.03	0.00	0.00	0.00	0.02
S.43	S	T1	0.02	0.33	0.21	0.13	0.10	0.05	0.04	0.04	0.03	0.01	0.00	0.01	0.02
S.53	C1S	T1	0.04	0.29	0.23	0.13	0.09	0.06	0.04	0.04	0.03	0.00	0.00	0.00	0.02
S.58	C1S	T1	0.03	0.30	0.22	0.10	0.08	0.06	0.04	0.04	0.03	0.05	0.00	0.00	0.03
S.59	C1S	T1	0.04	0.31	0.22	0.13	0.10	0.06	0.04	0.05	0.02	0.00	0.00	0.00	0.02
S.62	C1S	T1	0.04	0.29	0.27	0.11	0.09	0.07	0.04	0.03	0.02	0.00	0.01	0.00	0.02
S.52	C2S	T1	0.02	0.33	0.21	0.12	0.10	0.06	0.04	0.05	0.03	0.00	0.00	0.01	0.02
S.60	C2S	T1	0.02	0.31	0.26	0.12	0.09	0.06	0.04	0.04	0.03	0.00	0.00	0.01	0.02
S.61	C2S	T1	0.02	0.31	0.21	0.13	0.10	0.06	0.04	0.06	0.03	0.00	0.00	0.01	0.02
S.64	C2S	T1	0.02	0.30	0.23	0.14	0.10	0.06	0.04	0.05	0.03	0.00	0.00	0.01	0.02

Tab. S4 Relative abundance (%) at family level per microbial taxa and sample at T1. CK = no plant. no compost. B = Brassica. S = Sorghum. C1 = compost 1. C1B/C1S = Brassica/Sorghum with compost 1. C2 = compost 2. C2B/C2S = Brassica/Sorghum with compost 2.

sample	Experimental condition	Sampling time	Other	Sphingomonadaceae	Beijerinckiaceae	Pirellulaceae	Xanthobacteraceae	Gemmatimonadaceae	Rubrobacteriaceae	Bacillaceae	Solirubrobacteraceae	Gemmataceae	Nocardioidaceae	Geodermatophilaceae
S	CK	T0	0.68	0.05	0.04	0.02	0.03	0.02	0.02	0.01	0.04	0.02	0.04	0.03
S	CK	T0	0.65	0.06	0.05	0.02	0.03	0.02	0.03	0.01	0.04	0.02	0.04	0.03
SC1	C1	T0	0.69	0.06	0.04	0.02	0.02	0.02	0.02	0.02	0.02	0.02	0.04	0.03
SC1	C1	T0	0.71	0.05	0.04	0.02	0.02	0.02	0.02	0.03	0.02	0.02	0.04	0.03
SC1	C1	T0	0.69	0.06	0.04	0.02	0.02	0.02	0.02	0.02	0.03	0.02	0.03	0.02
SC2	C2	T0	0.69	0.06	0.04	0.02	0.02	0.02	0.02	0.02	0.03	0.02	0.03	0.02
SC2	C2	T0	0.67	0.07	0.04	0.02	0.03	0.02	0.02	0.01	0.03	0.02	0.04	0.03
SC2	C2	T0	0.68	0.06	0.04	0.02	0.02	0.02	0.03	0.02	0.03	0.02	0.03	0.02
S.65	CK	T1	0.72	0.03	0.04	0.04	0.03	0.03	0.02	0.02	0.02	0.02	0.01	0.02
S.67	CK	T1	0.73	0.02	0.03	0.03	0.03	0.03	0.03	0.02	0.03	0.02	0.01	0.02
S.70	CK	T1	0.70	0.02	0.04	0.04	0.03	0.03	0.02	0.03	0.03	0.03	0.01	0.02
S.9	B	T1	0.71	0.03	0.04	0.03	0.02	0.03	0.03	0.03	0.02	0.01	0.02	0.03
S.11	B	T1	0.71	0.04	0.04	0.03	0.03	0.03	0.03	0.02	0.02	0.02	0.01	0.02
S.12	B	T1	0.71	0.04	0.04	0.03	0.02	0.04	0.03	0.02	0.02	0.02	0.01	0.02
S.13	B	T1	0.69	0.04	0.04	0.04	0.03	0.03	0.03	0.02	0.02	0.02	0.02	0.02
S.33	S	T1	0.69	0.04	0.04	0.04	0.03	0.03	0.02	0.02	0.02	0.03	0.02	0.02
S.35	S	T1	0.71	0.04	0.03	0.04	0.03	0.02	0.02	0.02	0.02	0.03	0.02	0.02
S.36	S	T1	0.71	0.03	0.03	0.04	0.03	0.03	0.02	0.02	0.02	0.03	0.02	0.02
S.43	S	T1	0.69	0.04	0.04	0.04	0.03	0.03	0.02	0.02	0.02	0.03	0.02	0.02
S.73	C1	T1	0.71	0.03	0.03	0.04	0.03	0.03	0.02	0.04	0.02	0.02	0.01	0.02
S.75	C1	T1	0.72	0.02	0.03	0.04	0.03	0.03	0.03	0.03	0.02	0.02	0.01	0.02
S.76	C1	T1	0.73	0.03	0.03	0.04	0.03	0.03	0.02	0.03	0.01	0.02	0.01	0.02
S.21	C1B	T1	0.70	0.04	0.04	0.03	0.03	0.03	0.03	0.03	0.02	0.02	0.01	0.02
S.22	C1B	T1	0.73	0.04	0.03	0.03	0.03	0.03	0.02	0.02	0.02	0.02	0.01	0.02
S.23	C1B	T1	0.72	0.03	0.04	0.03	0.03	0.03	0.03	0.02	0.02	0.02	0.01	0.02
S.24	C1B	T1	0.73	0.03	0.04	0.03	0.02	0.03	0.02	0.03	0.02	0.02	0.01	0.02
S.53	C1S	T1	0.67	0.05	0.03	0.04	0.03	0.03	0.02	0.03	0.03	0.02	0.03	0.02
S.58	C1S	T1	0.69	0.07	0.04	0.03	0.02	0.02	0.02	0.02	0.02	0.02	0.03	0.02
S.59	C1S	T1	0.69	0.04	0.04	0.04	0.04	0.02	0.02	0.03	0.02	0.02	0.02	0.02
S.62	C1S	T1	0.66	0.06	0.05	0.03	0.02	0.03	0.03	0.03	0.02	0.02	0.03	0.02
S.78	C2	T1	0.72	0.03	0.04	0.05	0.03	0.03	0.02	0.02	0.01	0.02	0.01	0.02
S.80	C2	T1	0.73	0.03	0.04	0.04	0.04	0.03	0.02	0.02	0.01	0.02	0.01	0.01
S.82	C2	T1	0.70	0.05	0.04	0.03	0.03	0.03	0.02	0.02	0.02	0.02	0.01	0.03
S.29	C2B	T1	0.71	0.04	0.03	0.03	0.03	0.03	0.02	0.03	0.02	0.02	0.02	0.02
S.30	C2B	T1	0.71	0.03	0.03	0.03	0.03	0.03	0.02	0.03	0.03	0.02	0.02	0.02
S.31	C2B	T1	0.70	0.04	0.04	0.04	0.03	0.03	0.01	0.03	0.02	0.02	0.02	0.02
S.32	C2B	T1	0.69	0.05	0.04	0.03	0.03	0.03	0.02	0.03	0.02	0.02	0.02	0.02
S.52	C2S	T1	0.71	0.05	0.04	0.04	0.03	0.02	0.02	0.01	0.02	0.02	0.02	0.02
S.60	C2S	T1	0.66	0.08	0.05	0.03	0.03	0.03	0.02	0.01	0.02	0.02	0.03	0.02
S.61	C2S	T1	0.71	0.05	0.04	0.04	0.03	0.03	0.02	0.01	0.02	0.02	0.02	0.01
S.64	C2S	T1	0.71	0.06	0.03	0.04	0.03	0.03	0.02	0.01	0.02	0.02	0.02	0.01
